# Identification of the Collagen Types Essential for Mammalian Breast Acinar Structures

**DOI:** 10.3390/gels8120837

**Published:** 2022-12-18

**Authors:** Chandler R. Keller, Kelsey F. Ruud, Steve R. Martinez, Weimin Li

**Affiliations:** 1 Department of Translational Medicine and Physiology, Elson S. Floyd College of Medicine, Washington State University, Spokane, WA 99202, USA; 2 Department of Surgery, The Everett Clinic, Part of Optum, Everett, WA 98201, USA; 3 Providence Regional Cancer Partnership, Providence Regional Medical Center, Everett, WA 98201, USA; 4 Department of Medical Education and Clinical Sciences, Elson S. Floyd College of Medicine, Washington State University, Spokane, WA 99202, USA

**Keywords:** collagen, acini, extracellular matrix (ECM), hydrogel, breast cancer, 3D culture

## Abstract

Modeling human breast tissue architecture is essential to study the pathophysiological conditions of the breast. We report that normal mammary epithelial cells grown in human breast extracellular matrix (ECM) hydrogel formed acini structurally similar to those of human and pig mammary tissues. Type I, II, III and V collagens were commonly identified in human, pig, and mouse breast ECM. Mammary epithelial cells formed acini on certain types or combinations of the four collagens at normal levels of breast tissue elasticity. Comparison of the collagen species in mouse normal breast and breast tumor ECM revealed common and distinct sets of collagens within the two types of tissues. Elevated expression of collagen type I alpha 1 chain (Col1a1) was found in mouse and human breast cancers. Collagen type XXV alpha 1 chain (Col25a1) was identified in mouse breast tumors but not in normal breast tissues. Our data provide strategies for modeling human breast pathophysiological structures and functions using native tissue-derived hydrogels and offer insight into the potential contributions of different collagen types in breast cancer development.

## 1. Introduction

Collagens are essential structural and functional ECM proteins in the connective tissues of mammals. Among the 28 types of collagens, type I (Col I), II (Col II), III (Col III), V (Col V) and XI (Col XI) are fibril-forming and are often heterogeneously organized into fibrils to support the main structures of the tissues [1,2]. Depending on tissue types, type I, II, or III collagen could dominate the structural proteins in a tissue, while the other collagen types participate in fine-tuning or reinforcing the fibril networks formed by the major collagens. The differences in the types of collagens in a tissue define the structural fibril thickness and architecture [3,4]. Thus, the ECM structures of a specific tissue are uniquely formulated by different types of collagens and other ECM proteins for the tissue cells to properly perform their biological functions. 

We previously showed that polymerized Col I, Matrigel, and mammalian breast tissue ECM-derived protein hydrogel exhibited distinct microstructural features and mechanical properties [5,6,7]. Mammary epithelial cells grown on the different matrices expressed distinct receptors in response to the matrix protein ligands and displayed unique phenotypes and responses to therapeutic drugs [5,7,8,9]. These observations indicate the importance of not only the structural but also the compositional properties of the tissue ECM in cell biological activities and pathophysiological conditions. Therefore, studying the biology of human tissue cells and modeling human disease conditions in vitro require a close mimicry of the structural and compositional properties of the corresponding human tissues. 

One of the most commonly used tissue culture systems for mimicking human breast tissue acini or tumoroid formation is growing human mammary epithelial cells or breast cancer cells on laminin-rich ECM (lrECM), such as Matrigel, resembling the basement membrane (BM). Matrigel contains mainly laminin and Col IV, with minimal amounts of other collagens [8,10]. While laminin has been considered a prerequisite for epithelial cells to form acini or clustering structures, our recent data on three-dimensional (3D) cultures using animal or human breast tissue ECM hydrogel illustrate the potential to induce acinar structures in a collagen-rich microenvironment in the absence of laminin. Supporting this notion, breast luminal epithelial cells were shown to form aberrant, improperly polarized acinar structures in Col I [11]. To identify the collagen types that could support acini formation in breast tissue ECM, we have defined the ECM protein composition of human breast tissues, compared with those of pig and mouse breast tissues. We then formulated the major collagen types in the native ECM for in vitro acini formation experiments. In addition, the differences of collagen types in mouse normal breast and breast tumor ECM were identified.

## 2. Results

### 2.1. Proteomic Comparison of Human Breast Tissue ECM and ECM Hydrogel 

Human tissue ECM and its derived matrices are ideal and ultimate substrata that can provide a native tissue microenvironment for human biology-relevant cell cultures. Thus, understanding the structural and compositional properties of human tissue ECM is crucial for biomedical and bioengineering research. We previously reported the microstructures of decellularized human breast tissue ECM and its hydrogel [6]. In this study, we compared the proteomic compositions of normal human breast tissue ECM (HB-ECM) and human breast tissue matrix gel (HB-TMG) extracted from the breast ECM in order to verify the preservation of the native ECM proteins in HB-TMG for close mimicry of breast tissue microenvironment and biologically relevant tissue cultures. The preparation of HB-ECM and HB-TMG as well as liquid chromatography-coupled tandem mass spectrometry (LC-MS/MS) analysis of the samples were performed as we previously reported [6,7,8], with modifications, and have been described in the methods. 

**Figure 1:**
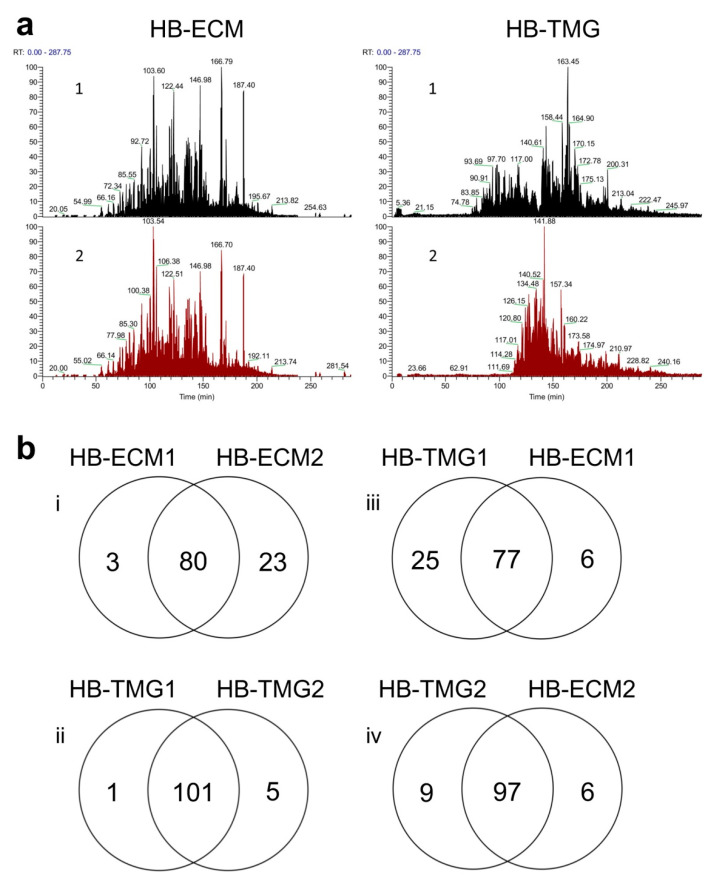
Proteomic comparison of HB-ECM and HB-TMG. (**a**) Mass spectrum peaks of the peptides corresponding to the identified proteins in HB-ECM and HB-TMG derived from two patient’s breast tissues. (**b**) Venn diagrams of the numbers of the common and the distinct proteins in the HB-ECM and the HB-TMG samples.

Our LC-MS/MS data showed that the majority of the mass peaks of the two HB-ECM and the two HB-TMG samples matched well (Figure 1a). The most mass peaks of the HB-TMG samples were also shown in the peaks for the HB-ECM samples (Figure 1a). The overlaps of the detected major proteins corresponding to the mass peaks of the different sample groups were analyzed and summarized as Venn diagrams (Figure 1b). High overlaps of the identified proteins between the two different HB-ECM samples (78%, Figure 1b-i) and between the two different HB-TMG samples (>95%, Figure 1b-ii) were observed. More than 92% of the HB-ECM proteins were identified in the HB-TMG extracts (Figure 1b-iii,iv). 

**Figure 2:**
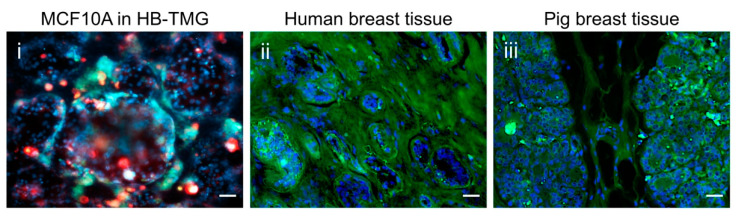
MCF10A cells formed acinar structures in HB-TMG that resemble those acini in human or pig breast tissues. (**i**) IF staining of E-cadherin (green); β4-integrin (red); nuclei (blue, Hoechst). (**ii**,**iii**) IF staining of the acinar structures in the cross sections of human or pig breast tissues. Green: collagen staining; blue: nuclei staining with Hoechst. Scale bars, 20 μm.

We previously reported that MCF10A human normal mammary epithelial cells formed acini structures when grown on top of ECM protein hydrogel extracted from pig breast tissue ECM (PB-ECM) or HB-ECM [5,6,7]. Since human breast acini are surrounded by intralobular stroma [12], we inspected the acini formation capacities of MCF10A cells embedded in HB-TMG (10,000 cells/uL of gel) at a concentration of 2 mg/mL, which is optimal for the gel to polymerize with an elastic modulus similar to that of normal human breast tissue ECM [6]. After 7–10 days of culture under optimal conditions, the cells formed clustered acinar structures (Figure 2i) resembling those seen in the lobules of human or pig mature normal breast tissues (Figure 2ii,iii). These data collectively indicate that HB-TMG preserves the majority of the ECM proteins of human breast tissues and supports structural organization of mammary epithelial cells.

### 2.2. The Common Collagen Types in Human, Pig and Mouse Breast ECM 

**Figure 3:**
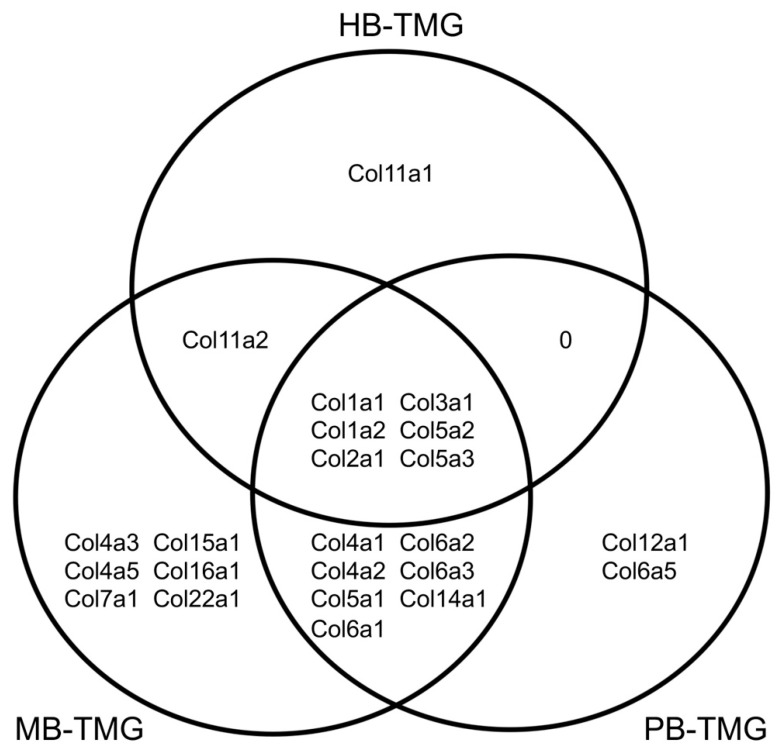
The common and different collagen types in HB-TMG, PB-TMG, and MB-TMG. The collagen chains in the three types of ECM hydrogels identified by LC-MS/MS were compared and clustered according to their presence or absence in the hydrogels.

Our previous work identified the protein compositions of mouse and pig breast tissue matrix (ECM) gels (MB-TMG/PB-TMG), respectively [7,8]. In this study, we defined the major proteins of HB-TMG. One of the prominent features common to MB-TMG, PB-TMG and HB-TMG compositions is the abundant overall collagen content. After comparing the collagen species of the three types of hydrogels (or their corresponding ECM), we found that Col1a1, Col1a2, Col2a1, Col3a1, Col5a2, and Col5a3 were commonly identified in the different types of gels (Figure 3). Additionally, Col11a2 was found in both HB-TMG and MB-TMG; Col4a1, Col4a2, Col5a1, Col6a1, Col6a2, Col6a3, and Col14a1 were found in both MB-TMG and PB-TMG. Interestingly, Col11a1 was only identified in HB-TMG; Col4a3, Col4a5, Col7a1, Col15a1, Col16a1, and Col22a1 were only found in MB-TMG; Col12a1 and Col6a5 were only found in PB-TMG. These data suggest that the three types of mammalian breast ECM all contain Col I, Col II, Col III, and Col V, with each type of the ECM having their unique collagen species.

### 2.3. Mammary Epithelial Acini Formation in Formulated Collagens

MB-TMG, PB-TMG and HB-TMG are all collagen-rich (70–90% of the major ECM proteins) and contain minimal amount of laminin (< 2% of the major ECM proteins), which is a key component of BM that supports acinar structures formed by epithelial cells within secretory gland-containing tissues, including breast. Since the breast ECM hydrogels support acini formation [5,6,7] (and Figure 2i) and Col I, Col II, Col III and Col V comprise the majority of mouse, pig, and human breast ECM proteins, we questioned whether any of these major collagens alone or in combination could support acini formation. 

To test this, we first analyzed the ratios of the common types of collagens in MB-TMG, PB-TMG and HB-TMG in order to formulate their abundance in composite hydrogels as they present in native ECM for the experiment. Our analysis suggested an abundance ratio of Col I:Col III:Col V:Col II at about 15:10:1:1. Based on this ratio and a final total collagen concentration of 2 mg/mL, which allowed the gels to polymerize with an elastic modulus similar to that of normal human breast tissue ECM [5,6], we reconstituted hydrogel solutions with either Col I, Col II, Col III, or Col V alone or their different combinations and performed acini formation assays by growing MCF10A cells on top of the polymerized gels coated at the bottom of the wells of 96-well plates.

**Figure 4:**
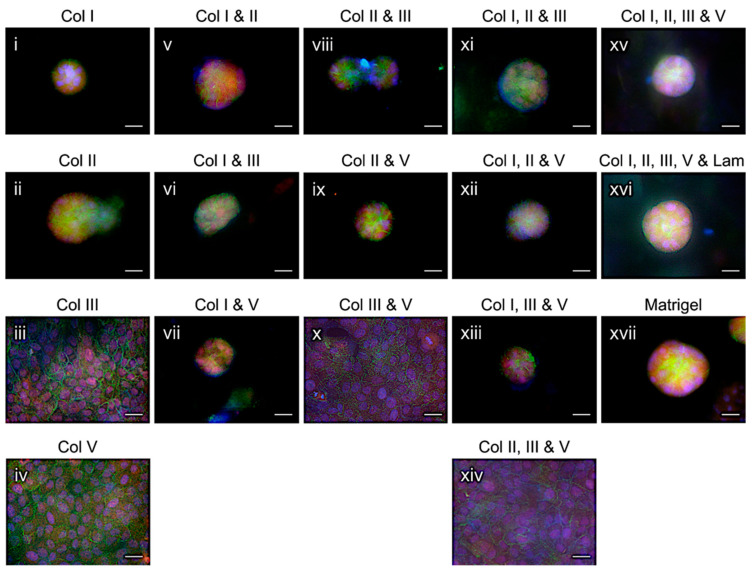
MCF10A cell acini formation on a single type or a combination of the different types of collagens commonly identified in HB-TMG, PB-TMG, and MB-TMG. IF staining was used to examine the acini formation on Col I (**i**); Col II (**ii**); Col III (**iii**); Col V (**iv**); Col 1& Col II (**v**); Col I & Col III (**vi**); Col I & Col V (**vii**); Col II & Col III (**viii**); Col II & Col V (**ix**); Col III & Col V (**x**); Col I, Col II & Col III (**xi**); Col I, Col II & Col V (**xii**); Col I, Col III & Col V (**xiii**); Col II, Col III & Col V (**xiv**); Col I, Col II, Col III & Col V (**xv**); Col I, Col II, Col III, Col V & laminin (**xvi**). Acini formation on Matrigel was included as a positive control (**xvii**). Green, E-cadherin; red, β4-integrin; blue, nuclei stained with Hoechst. Scale bars, 50 μM.

Our data showed that MCF10A cells formed acini structures on Col I (Figure 4i), Col II (Figure 4ii), Col I and Col II (Figure 4v), Col I and Col III (Figure 4vi), Col I and Col V (Figure 4vii), Col II and Col III (Figure 4viii), Col II and Col V (Figure 4ix), Col I, Col II and Col III (Figure 4xi), Col I, Col II and Col V (Figure 4xii), Col I, Col III and Col V (Figure 4xiii), and Col I, Col II, Col III and Col V (Figure 4xv). Addition of laminin (5%) to Col I, Col II, Col III and Col V mixture neither increased the sizes of the formed acini nor sped up the acini formation (Figure 4xvi). However, we observed that acini formation on the gels comprised of Col I, Col II, Col III and Col V was more consistent and robust, as seen in the Matrigel group (Figure 4xvii), than that on any single or other combined collagens, which were able to support acini formation. The cells grown on Col III (Figure 4iii), Col V (Figure 4iv), Col III and Col V (Figure 4x), or Col II, Col III and Col V (Figure 4xiv) failed to form acini. Since certain types of the collagen gels attached to the bottom of the tissue culture wells tended to lift over a long culturing time, a 5 to 7-day observation window was used. Because of the short culturing time, acini lumen formation was not observed. These data indicate that acini can form in certain fibrillar collagens or their combinations in the absence of a BM or the major proteins of BM.

### 2.4. Differences of Collagen Types in Normal Breast and Breast Tumor Tissue ECM

Abnormal collagen deposition in tumors and tumor-associated collagen signatures (TACS) have been suggested as markers of breast cancer progression [13,14]. We recently showed that the collagens within decellularized normal human breast tissue ECM displayed an organized mesh-like and fibrous architecture, whereas those in invasive ductal carcinoma (IDC) ECM exhibited disrupted and aggregated structures [6], suggesting collagen deposition and destruction changes in breast cancer. Yet, the variations of the types of collagens in breast tumors from those in normal breasts have hitherto not been defined. In this study, we extracted ECM proteins from mouse mammary tumors derived from human MDA-MB-231 breast cancer cell inoculation in mammary fat pad (MFP), followed by identification of the proteins with LC-MS/MS and comparison of the differences of the major collagens with those in normal breast ECM. 

**Figure 5:**
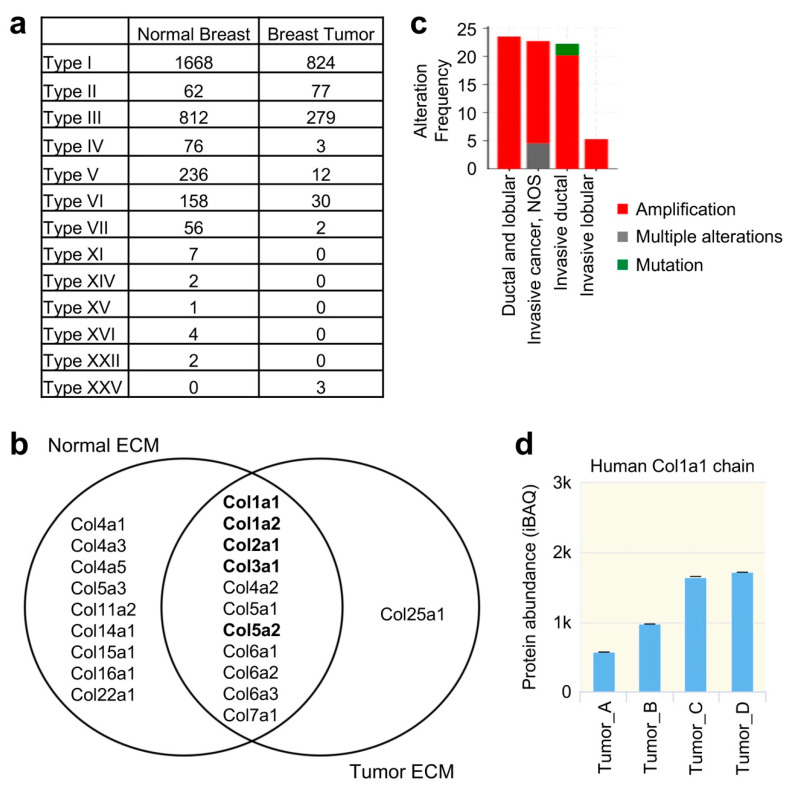
Collagen type and expression level differences between normal and breast tumor tissues. (**a**) A table showing the levels of the different types of collagens in mouse normal breast ECM and breast tumor ECM based on mass spectrometry analysis. (**b**) The common and distinct collagen chains identified in the normal breast ECM and breast tumor ECM. The collagen chains indicated in bold are the ones commonly found in MB-TMG, PB-TMG, and HB-TMG. (**c**) Col1a1 genetic alteration frequencies in invasive/metastatic human breast cancers. (**d**) Col1a1 protein expression increases in invasive human breast cancers.

We found that the levels of the major collagen types in the tumor ECM were overall less than those in normal ECM (Figure 5a). Col1a1, Col1a2, Col2a1, Col3a1, and Col5a2, which were commonly found in normal ECM of mouse, pig, and human breast tissues (Figure 3), were also identified in tumor ECM (Figure 5b) in addition to Col4a2, Col5a1, Col6a1, Col6a2, Col6a3, and Col7a1. On the other hand, Col4a1, Col4a3, Col4a5, Col5a3, Col11a2, Col14a1, Col15a1, Col16a1, and Col22a1 were found in normal ECM but not in tumor ECM; Col25a1 was found only in tumor ECM but not in normal ECM.

Heterotrimeric Col I (two α1 chains/Col1a1 and one α2 chain/Col1a2) is the most abundant type of collagen in the breast tissues [7,8], consistent with the levels of Col I in human breast [7,15]. Col I homotrimer [Colα1(I)_3_], which contains three α1 chains [16,17,18], is only found in fetal tissues, fibrosis, and cancers [19,20,21] that is thought to mediate cancer cell motility [22,23]. We found that the Col1a1 to Col1a2 ratio was 1.2 in mouse normal breast ECM and 2.6 in mouse breast tumor ECM, a more than two-fold increase in Col1a1 chain in the tumor ECM. This is consistent with our data-mining results from The Metastatic Breast Cancer Project (cBioPortal, Figure 5c) and The Proteomic Landscape of Triple Negative Breast Cancer database (zucchini.gs.washington.edu/BreastCancerProteome, Figure 5d), where high levels of Col1a1 genetic alterations, especially amplifications, or protein expression were found in invasive human breast cancer samples. These data collectively suggest that breast tumor ECM has the major structural collagen types as the normal breast ECM, with Col I homotrimers substantially replacing Col heterotrimers and alterations in minor ECM contents.

## 3. Discussion

Human breast tissue ECM is a collagen-rich framework supporting the 3D structures and functions of mammary acini. Defining the breast ECM proteins and the abundance of different types of collagens in the ECM is crucial for studying the structural and functional changes of human breast tissues under pathophysiological conditions. This and our previous works have reported the compositions of human, mouse [8], and pig [7] breast tissue ECM and their derived hydrogels, which are all biologically relevant tissue culture substrata highly capable of supporting mammary epithelial cells to display phenotypes as seen in native tissues [5,6,7,8]. The successful extraction of the majority of ECM proteins from breast tissue ECM and the generation of the ECM protein hydrogels (Figure 1a,b) have enabled the observations of mammary epithelial cell biology in natural tissue microenvironments resembling those of native tissues (Figure 2). This culture platform warrants further application in studying the spatial and temporal interactions of normal or cancerous epithelial cells with stromal cells as well as cell modifications of the ECM space initiated by cellular genetic changes or in response to microenvironment stimuli, such as growth hormones or tumorigenic signals. We also envision an increased use of native ECM or their-derived hydrogel systems in advanced biomedical or bioengineering applications because of their advantageous structural and compositional nature compared to synthetic tissue culture substrata [24].

Comparison of HB-TMG with MB-TMG and PB-TMG revealed the common types of collagens in human, mouse, and pig mammary tissues (Figure 3). Interestingly, the collagens common for the breast ECM of human, mouse, and pig are the fibrillar Col I, Col II, Col III and Col V, which are the main mechanical support, cellular receptor ligands and structural organizers of ECM [25]. These essential structural ECM collagens are important for native mammary epithelial cell functional differentiation, which requires interactions between the cells and the ECM substratum [26]. The existence of the distinct collagen species in the mammary tissues of the three different mammalian species likely associates with the intrinsic nature of the mammary glands, such as genetic background, morphogenesis, involution, structures and functions, in the individual mammals. 

So far, most of the acini formation assays have been performed on BM substitutes, such as laminin-rich ECM (lrECM) or Matrigel. While this model system is robust in producing acinar structures in tissue cultures, the growth hormones and other underdefined intracellular biomolecules contained in the type of hydrogel derived from Engelbreth-Holm-Swarm (EHS) mouse sarcomas potentially interfere with the signaling, biological functions, phenotypes and, thus, the responses of the cells cultured on the gel to treatment [8,27,28]. It is therefore necessary to have alternative hydrogel models that also carry native ECM structural and compositional properties, but less compositional noise backgrounds for acini modeling and other biologically relevant studies. In addition to HB/PB/MB-TMG, which supports acini formation in tissue cultures [5,6], Col I was reported to facilitate breast luminal epithelial cells forming acini [11]. Consistently, we observed MCF10A cells formed acini in Col I (Figure 4i). It is interesting that the cells also formed acini on Col II (Figure 4ii), a predominant component of cartilage, and a set of hydrogels composed of different combinations of Col I, Col II, Col III, and Col V (Figure 4v–ix,xi–xiii,xv). These findings clearly provide additional options for modeling acinar structures in a known and formulable ECM microenvironment. Since mammary luminal epithelial cells, which have limited contacts with the BM, express high levels of laminin α5 (a type of BM laminin) that can be deposited on the BM and myoepithelial cells secrete laminin α1, α3 and α5 [29], epithelial cell and myoepithelial cell co-culture in formulated collagens, as tested by Gudjonsson et al. in Col I [11], will add novel insights into modeling human mammary gland and regulation of acinar cell functions under pathophysiological conditions. Moreover, ECM proteins other than the fibrillar collagens can be blended into the collagens that support acini formation to study their regulation of cell biology or cell-ECM protein interactions. 

In addition to the compositional capacities of the major ECM collagens in mediating acini formation, the mechanical properties of the collagens could potentially regulate not only the formation, but also the sizes and shapes of the acini structures [30]. Based on the elastic moduli of porcine or human breast tissue ECM [6,7], this study has used collagen hydrogels at the working concentration of 2 mg/mL, which is the optimal concentration for the collagen-rich hydrogels derived from the native ECM to polymerize to the ECM’s stiffness [5], for the acini formation assays. Future investigations in the effects of the stiffness and microstructure alterations of the different collagen gels on acini formation will add further insights into the pathophysiological conditions of the mammary gland.

Breast malignancy and progression are accompanied by destruction of the tissue ECM architecture, excessive collagen deposition, and desmoplastic changes [31,32]. It was reported that collagen fiber density is positively associated with local mammographic density of the breast and may serve as markers for cancer risk and progression [33]. Indeed, increased Col I deposition in breast tumors can cause tissue hardening, increased cancer cell proliferation, migration, tumor angiogenesis, and tumor-associated macrophage recruitment [30,31,34,35,36,37]. One understudied aspect of collagen deposition in tumors is whether the collagens are structurally and compositionally polymerized correctly. In this study, while we observed that the species of the major collagens, such as Col I, Col II, Col III and Col V, remained unchanged (Figure 5b), certain collagen chains existing in normal breast ECM disappeared in breast tumor ECM (Figure 5b) and increased Col1a1 gene expression and protein levels were identified in breast tumors (Figure 5c,d). This is consistent with augmented Col1a1 expression in cancer cells [38,39] that associates with high rates of metastasis and poor survival [40]. Additionally, the increased Col1a1 to Col1a2 ratio in tumors suggests either an overproduction or an enhanced integration of Col1a1 into the Col I fibers, which become homotrimeric Col1a1-rich. Given that both cancer cells and cancer-associated fibroblasts (CAFs) are possible producers of excessive ECM collagens [19,22,41], future studies using in vivo-mimicking 3D co-cultures of cancer cells and fibroblasts coupled with gene expression and proteomics analyses for de novo collagen generation and related regulating nexus will shed light on the mechanisms and control of ECM-mediated tumor progression [8,42].

In conclusion, our data indicate that Col I, Col II, Col III and Col V are the building blocks of both normal breast and breast tumor ECM. These types of collagens, either alone or in combination, can serve as basic matrix supports for breast acini modeling. The collagen species and chain type alterations in tumors promise new formulations of tissue culture substrata and novel ECM markers for cancer diagnosis, prognosis, and therapeutic targeting.

## 4. Materials and Methods

### 4.1. Patient Specimens

Fresh breast tissues from two patients having invasive ductal carcinoma and undergoing mastectomy were processed, following the protocol approved by our Institutional Review Board (IRB; STUDY2019000160 for The Everett Clinic site and exempted for the Washington State University site) as we previously reported [6], for histological staining, ECM extraction, and hydrogel generation. 

### 4.2. Human Breast Tissue ECM Extraction

This was performed as we reported [6]. Briefly, the breast tissues were diced, homogenized in ice cold deionized water, decellularized in 1% Triton X-100 at RT for 5–7 days, washed in deionized water for at least 3 times to remove the detergent. The decellularized ECM was freeze-dried and stored for hydrogel extraction.

### 4.3. Hydrogel Generation from Breast Tissue ECM

HB-TMG hydrogel was produced following our established protocol as reported [6].

### 4.4. Identification of Human Breast Tissue ECM Proteins

Enzymatic “In Liquid” Digestion of ECM: ECM powder extract was partially solubilized in 1.5 mL tube with 1 mL of 8 M Urea containing 0.1% ProteaseMAX (Promega) and 50 mM NH_4_HCO_3_ (pH 8.5). Samples were sonicated twice for 30 s each with a sonication probe, cooled on ice in between, then stored overnight at 4 °C to facilitate further solubilization. Another round of sonication as previously described was executed and 40 µL of homogenously reconstituted samples was taken for the downstream 200 µL digestion, where sample was diluted to 120 μL for reduction step with: 5 μL of 25 mM DTT and 75 μL 25 mM NH_4_HCO_3_ (pH 8.5). Incubated at 56 °C for 15 min, cooled on ice to room temperature then 6 μL of 55 mM CAA (Chloroacetamide) was added for alkylation and incubated in darkness at room temperature for 15 min. Reaction was quenched by adding 16 μL of 25 mM DTT. For protease digestion, 30 μL of Trypsin/LysC mix solution [50 ng/μL *Trypsin* from PROMEGA and 50 ng/μL *LysC* from WAKO in 25 mM NH_4_HCO_3_] and 28 μL of 25 mM NH_4_HCO_3_ (pH 8.5) was added to 200 µL final volume. The digestion was conducted at 37 °C overnight and terminated by acidification with 2.5% Trifluoroacetic Acid (TFA). ProteaseMAX was removed by centrifugation (maximum speed) at room temperature for 10 min.

NanoLC-MS/MS: Digested samples were cleaned up using OMIX C18 SPE cartridges (Agilent, Palo Alto, CA, USA) per manufacturer protocol and eluted in 20 µL of 60/40/0.1% ACN/H_2_O/TFA, dried to completion in the speed-vac and reconstituted in 80 µL of 0.1% formic acid and 2% acetonitrile. Peptides were analyzed by nanoLC-MS/MS as we previously reported [8].

Data analysis: Raw MS/MS data were converted to mgf file format using MSConvert (ProteoWizard: Open Source Software for Rapid Proteomics Tools Development). The resulting mgf files were used to search against *Homo sapiens* amino acid sequence database with decoy reverse entries and a list of common contaminants (97,184 total entries from UniProt UP5640 database downloaded 8 November 2020) using in-house *Mascot* search engine 2.7.0 (Matrix Science) with fixed cysteine carbamidomethylation plus variable Methionine, Proline oxidation and Asparagine, Glutamine deamidation. Peptide mass tolerance was set at 10 ppm and fragment masses at 0.6 Da. Protein annotations, significance of identification and spectral based quantification was done with help of Scaffold software (version 4.11.0, Proteome Software Inc., Portland, OR, USA). Protein identifications were accepted if they could be established at greater than 99.0% probability within 1% False Discovery Rate and contained at least 2 identified peptides. Protein probabilities were assigned by the Protein Prophet algorithm [43].

Data availability: The mass spectrometry proteomics data for human breast tissue ECM/hydrogel and mouse breast tumor tissue ECM/hydrogel have been deposited to the ProteomeXchange Consortium via the PRIDE partner repository with the data sets’ identifiers PXD037900 and PXD037920, respectively. The previously reported mouse breast tissue ECM/hydrogel and pig breast tissue ECM/hydrogel proteomics data set identifiers are PXD005697 and PXD011011, respectively.

### 4.5. Cell Culture

MCF10A human mammary epithelial cells (ATCC) were cultured (37 °C, 5% CO_2_) in DMEM/F12 containing 5% horse serum, 20 ng/mL EGF, 1% penicillin/streptomycin, 0.5 μg/mL hydrocortisone, 100 ng/mL cholera toxin, and 10 μg/mL human insulin.

### 4.6. Acini Formation

MCF10A cells were resuspended in HB-TMG (2 mg/mL, 20,000 cells in 2 μL of hydrogel) and spotted at the bottom of the wells of 96-well plates. After gel polymerization in a 37 °C incubator (supplied with 5% CO_2_) for about 30 min. Cell culture medium (200 μL/well) was added to the wells. The encapsulated cells were cultured (37 °C, 5% CO_2_) and observed for acini formation in 10 days. For acini formation on Col I (porcine, Chondrex, Inc., Woodinville, WA, USA), Col II (bovine, MD Bioproducts, Zurich, Switzerland), Col III (human, BioVision, Milpitas, CA, USA), or/and Col V (bovine, Advanced BioMatrix, Carlsbad, CA, USA), 20 μL of the individual collagens either alone or in combination (Figure 4), with a final total concentration of 2 mg/mL, were coated at the well bottom of a 96-well plate. Since we noticed differences in the times needed for the different collagen gels to fully polymerize, the coated gels were allowed to polymerize in a humidified incubator (37 °C, 5% CO_2_) overnight. The temperature used for gel polymerization was based on human normal body temperature. Other temperatures that may allow the gels to polymerize at different velocities were not tested in this study and can be studied separately. After gel polymerization, 2000 MCF10A cells were seeded into the collagen-coated wells and cultured as described above for 5–7 days. Laminin (5%, ThermoFisher Scientific) was added to a Col I, Col II, Col III and Col V mixture as indicated in Figure 4. The cultures were fixed with paraformaldehyde, permeabilized, and incubated with primary antibodies against E-cadherin (Thermo Fisher Scientific, Waltham, MA, USA) and β4 integrin (R&D Systems, Minneapolis, MN, USA), followed by fluorophore-conjugated secondary antibodies and Hoechst staining and imaging for acinar structures under a fluorescent microscope. 

### 4.7. Immunofluorescence (IF) Staining of FFPE Samples

IF staining was performed as we reported [6].

## Data Availability

All data are available in the manuscript.
